# Materials for Infectious Diseases

**DOI:** 10.3390/ijms24043295

**Published:** 2023-02-07

**Authors:** Ali Zarrabi, Fabrizio Caldera, Francesco Trotta

**Affiliations:** 1 Department of Biomedical Engineering, Istinye University, Istanbul 34396, Turkey; 2 Department of Chemistry, University of Turin, Via Pietro Giuria 7, 10125 Torino, Italy

The COVID-19 pandemic showed the crucial significance of investing in and conducting research on infectious diseases. This research is not limited to introducing new antimicrobial agents; it also includes rethinking old antimicrobial agents by combining them with new technologies, such as nanotechnology, developing rapid, reliable, and point-of-care diagnosis techniques, and introducing prevention techniques. The common part of all of this research is the specific materials used in prevention/diagnosis/treatment formulations. 

For prevention purposes, antimicrobial textiles containing ascorbic acid and spunlace greige cotton nonwoven fabrics were developed as affordable fabrics for producing face masks and wound dressings. These textile showed the “complete inhibition of both Gram-negative and Gram-positive bacteria activity commensurate with levels necessary for commercial use” [1]. In another work, it was shown that adding antimicrobial nanoparticles to a tissue replacement structure could result in a nanocomposite with antibacterial features. Therein, zinc and graphene were added to hydroxyapatite; the resultant nanocomposite demonstrated 2.7 and 3.4 times more antibacterial properties than hydroxyapatite nanoparticles [2]. In addition to being used in nanocomposites, graphene oxide was introduced as an antibacterial material against various types of bacteria [3].

In the context of diagnoses, there are papers, such as the research conducted by Adamczyk et al., which describe the importance of investigating the mechanism of the “non-specific adsorption of immunolatex particles at solid substrates” with different charges. This study could be useful for designing more efficient biosensors [4]. As a point-of-care sensor to detect dilute biomarkers, Edwards and colleagues fabricated an optical protease sensor based on peptide–cellulose conjugates. They demonstrated the potential of nanocellulose in improving sensor biocompatibility as well as sensitivity through its high specific surface area properties [5].

For treatment purposes, we developed water-soluble β-cyclodextrin-based anionic polymers and tested them against herpes simplex virus (HSV-2) as well as respiratory syncytial virus (RSV). Our results showed that “polymer virucidal activity against RSV can be exploited to produce new antiviral materials” [6]. In another work, Rozga-Wijas and coworkers synthesized a water-soluble smart nanoparticle composed of phenosafranin–polyhedral oligomeric silsesquioxane (POSS) conjugates with photodynamic antibacterial potential against Gram-positive and Gram-negative bacteria. They demonstrated that, upon the irradiation of the covalent conjugates, reactive oxygen species (ROS) are generated. These ROS can eradicate bacterial cells, leading to the death of bacteria [7]. In another paper, the exceptional role of engineered nanoparticles in efficient drug/vaccine delivery against viral diseases was covered, as was “a perspective view of the vaccines and nano-vaccines development against the COVID-19 pandemic” [8].

With reference to the mentioned topics, the aim of this Special Issue is to highlight the benefits of emerging/advanced materials in different aspects of infectious diseases, covering prevention, diagnosis, and treatment, as well as the features that materials should bear in order to enter clinical trials.
